# The impact of using peer interviewers in a study of patient empowerment amongst people in cancer follow‐up

**DOI:** 10.1111/hex.12655

**Published:** 2017-12-05

**Authors:** Clara R. Jørgensen, Nanna B. Eskildsen, Thora G. Thomsen, Inger D. Nielsen, Anna T. Johnsen

**Affiliations:** ^1^ Warwick Medical School University of Warwick and School of Education University of Birmingham Birmingham UK; ^2^ Department of Psychology University of Southern Denmark Odense Denmark; ^3^ Roskilde and Koege Hospitals Roskilde Denmark; ^4^ University of Southern Denmark Odense Denmark; ^5^ PPI Representative Copenhagen Denmark; ^6^ Department of Palliative Medicine Bispebjerg Hospital Copenhagen Denmark

**Keywords:** cancer research, empowerment, patient and public involvement, peer interviewers, qualitative interviews

## Abstract

**Background:**

A range of benefits have been reported from engaging peer interviewers in qualitative interviews, but little systematic evaluation exists to assess their impact on both process and outcomes of qualitative interviews in health research.

**Objective:**

To investigate the impact of involving patient representatives as peer interviewers in a research project on patient empowerment.

**Design:**

18 interviews were carried out as part of the wider study, seven by the academic researcher alone and eleven jointly with a peer interviewer. The interviews were analysed quantitatively and qualitatively to explore potential differences between interviews conducted by the researcher alone and interviews conducted jointly by the researcher and the peer interviewers. A phone evaluation of the peer interviews was carried out with the research participants, and notes were thematically analysed to understand their experiences.

**Results:**

Differences were identified between the academic researcher and the peer interviewers in the types of questions they asked and the degree to which personal narrative was used in the interview. Peer interviewers varied significantly in their approach. Research participants were positive about the experience of being interviewed by a peer interviewer. No firm conclusions could be made about impact on outcomes.

**Discussion and conclusions:**

The uniqueness and complexity of qualitative interviews made it difficult to provide any firm conclusions about the impact of having peer interviewers on the research outcomes, and the benefits identified from the analysis mostly related to the process of the interviews. Benefits from using peer interviewers need to be considered alongside relevant ethical considerations, and available resources for training and support.

## 1 INTRODUCTION

This paper explores the impact of involving patient representatives as peer interviewers in a Danish research project on patient empowerment amongst people in cancer follow‐up (from now on “the Empowerment study”). The Empowerment study is a three‐year (2015‐2017) mixed‐method project, which has explored the multiple aspects of patient empowerment amongst Danes in cancer follow‐up, using semi‐structured interviews^1^ and questionnaires. The study draws on Zimmerman's^2^ and Rappaport's^3^, ^4^ understandings of empowerment as a process by which patients develop knowledge, skills and motivation to take control of their own situation and the state in which they have a sense of being in control or having mastery. Empowerment is a concept which has grown in importance in cancer care, as people are living for longer periods of time with the illness and increasingly given responsibility for their own care.^5^ As one of the first projects of its kind in Denmark, the Empowerment study incorporated Patient and Public Involvement (PPI) into the research process from its beginning. As part of this, the study involved people with experience of cancer as peer interviewers in the qualitative semi‐structured interviews.

Peer interviewers are generally understood as people who have “direct experience of the topic being researched” and carry out interviews with research participants, who have similar experiences.^6^ The benefits reported from this process are that the shared experiences of peer interviewers and research participants may facilitate access to groups that are hard to reach,^7^ minimize potential power imbalances,^8^ and help research participants feel more relaxed, open and honest.^9^, ^10^ Peer interviewers may be able to strike up a better rapport with participants^11^, ^12^ and may furthermore be able to bring new and different perspectives to the interviews due to their own personal experience of the topic being researched.^13^ However, researchers have also acknowledged some of the potential challenges of peer interviews, particularly the relative inexperience of peer interviewers of interview techniques, the potential distress caused by interviewing people in similar situations and the logistics and time involved.^8^, ^12^, ^14^

Both benefits and challenges of peer interviews are most often described from the perspectives of researchers and peer interviewers, for example. 15, 16, 17, 18 Not much is known about how interviewees experience peer interviews and whether they feel that it makes a difference to the interview. Furthermore, the literature predominantly focuses on the impact of peer interviewers on the process of the interview, and less on the impact on research outcomes. Gillard et al^19^ have attempted to develop a systematic and methodologically robust approach to evaluate the impact of service user researchers on the research process but no similar attempts to assess impact on research outcomes have been identified.

The present paper explores the impact of involving peer interviewers in the Empowerment study, considering various perspectives, focusing on both process and outcomes and using a mixture of methods. The paper is based on data collected through 18 semi‐structured interviews with 16 people in cancer follow‐up, conducted in the period September 2015‐March 2016. Eleven of the interviews were carried out by the main academic project researcher and a peer interviewer and seven of them by the academic project researcher alone. In the paper, we compare the two types of interviews and seek to answer the following two questions:

1. Were there any differences in the way the academic researcher and the peer interviewers conducted the interview (process)?
2. Were there any differences in the themes and topics discussed in the two types of interviews (outcomes)?

## 2 PPI IN THE EMPOWERMENT Study

PPI was integrated into the design of the Empowerment study from its very beginning. Before the submission of the research proposal, a group of four patient representatives were invited to a workshop to discuss the proposal. After funding had been obtained, a steering group was set up, including two patient representatives, the two main researchers, and the co‐applicants on the project. In addition, seven co‐researchers were recruited. No particular criteria were set for applying, except for having had experience of cancer either personally or as a relative, and having an interest in research.

The co‐researcher group met for the first time in May 2015, where they completed a four‐day training course, facilitated by two project researchers, including material on what it means to be an involved user, research methods, reflexivity and ethics. Another training day was facilitated in August 2015, where participants were trained specifically in interview techniques and carried out practice interviews with each other. They were presented with a draft interview guide for the qualitative interviews, which they discussed in the group and revised together with the project researchers. The qualitative interviews for the project commenced shortly after, and of the nine co‐researchers, five became involved as peer interviewers carrying out between one and four interviews each.

## 3 THE QUALITATIVE INTERVIEWS

The qualitative stage of the Empowerment study consisted of 18 interviews, conducted with 16 different people in cancer follow‐up, either by the academic researcher alone (referred to as ARA interviews) or jointly by the academic researcher and the peer interviewer (referred to as API interviews) (Table 1).

Peer interviews are often conducted without an academic researcher present, but in our particular study, it was decided to include the academic researcher, so that she could provide support if needed and be able to debrief with the peer interviewers afterwards.

From the beginning of the study, the qualitative interviews were considered both as a data‐collection tool, enabling the research team to explore the overall research questions of the Empowerment study and form the basis for the development of a PROM questionnaire^1^ and as an exercise to explore the impact of peer interviewing. Both interviewees and peer interviewers had given their informed consent, considering their respective roles in the study of patient empowerment and peer interviewing.

The peer interviewers had been involved in the development of the semi‐structured interview guide as part of their training, and were explicitly informed that they could ask outside the question guide if they felt the need.

The interviews were recorded and transcribed verbatim by two student assistant who had been given a protocol for the transcription to assure consistency, but did not know at the time that the transcripts would be used to analyse both patient empowerment and the impact of peer interviewers. The transcripts were analysed separately for the two purposes.

### 3.1 The interviews with participants evaluating their experience

The interviewees who had been interviewed by a peer had all been asked whether they agreed to be phoned shortly after the qualitative interview and asked about their experiences of the interview. A semi‐structured question guide was constructed in which they were asked about their general experiences of the interview, what they had initially thought about the idea of having a peer interviewer present, how they had felt about it during and after the interview and whether they thought it had made a difference. All eleven of them were interviewed over the phone by a different researcher than the one who had been part of the interviews. Extensive notes were taken during the interview, revised and expanded directly after the interview, and included as data in our assessment of the impact of peer interviews seen from the perspective of interviewees.

## 4 EVALUATING THE IMPACT OF PEER INTERVIEWERS

To explore the impact of having peer interviewers conducting the qualitative semi‐structured interviews, a three‐stage data analysis was conducted, including (i) a quantitative analysis of the interview transcripts inspired by Gillard et al's approach,^19^ but extended to include more measures (ii) a qualitative analysis of the interview transcripts and (iii) a content analysis of notes from phone evaluations with interviewees.

### 4.1 Stage 1: The quantitative analysis of transcripts

The quantitative analysis of the interview transcripts involved four elements: first, the amount of words spoken by each participant in the interviews and the amount of questions and comments (entries) posed by the academic researcher and the peer interviewers, respectively, were counted and compared. Second, the entries were divided into nine different categories, based on previous readings of the transcripts: (i) Questions, (ii) Confirming comments (eg yes, mm.), (iii) Short non‐confirming comments (eg “Really?” “Are you sure?”) (iv) Personal narrative, attitudes, thoughts or experiences, (v) Advice, (vi) Answering questions, (vii) Wondering/querying comments (eg “well,” “hmm”), (viii) Small talk or meta talk (eg “ok, let's move on”) and (ix) Reflection on what had been said, continuation of sentences or repetition of what the interviewee had said.

Third, the questions posed by the academic researcher and the peer researcher in the interviews were coded using nine thematic codes, which had been identified through an earlier broad coding of the transcript: (i) Individual situation, for example personality, everyday life, individual circumstances, co‐morbidity. (IND), (ii) Health‐care providers, incl. trust, access, and continuity (HCP), (iii) Information, including level, timing and access (INF), (iv) Knowledge, skills and body awareness (KSB), (v) Illness journey (ILJ), (vi) Decision‐making and actions (DMA), (vii) Economic situation (ECO), (viii) Social networks (SOC) and (ix) The system, for example waiting lists, communication between different departments . (SYS). Fourth, the text as a whole was coded using the same nine thematic codes, allowing for a count and comparison of the average amount of words used on each code in the different types of interviews.

In exercise three and four, some of the text and questions were coded more than once, due to them being relevant to more than one thematic code. Some of the text was also not coded, if not relevant to the thematic codes (if for example it was about having a cake or answering the phone). As a result, the amount of questions or words that came out of adding the nine thematic codes together in exercise three and four was sometimes higher or lower than the initial amount of words or comments figuring in exercise one and two, where all words were counted, and only once.

### 4.2 Stage 2: Qualitative analysis of transcripts

The interview transcripts were read and analysed using a qualitative approach, which identified relevant text, repeated ideas and themes^20^ in our attempt to identify if and how the academic researcher and the peer interviewer used personal information and personal knowledge of a given topic in the interviews, whether it appeared to make any difference to the interview, and whether there were any other differences between the two types of interviews, for example in relation to the dynamics in the interviews or the way things were being said. Notes were made of exchanges within the interviews that illustrated particular dynamics or themes and at the end, a few sentences were written to summarize the overall impression of the interview. These were subsequently compared to see whether any significant differences could be found in the processes and outcomes of the interviews.

### 4.3 Stage 3: Analysis of phone interviews with participants

The notes from the phone conversations with interviewees were read and analysed, again identifying repeated ideas and common themes in their views on being interviewed by a peer interviewer, their experiences of the process and whether they had felt that it had made a difference to them or the outcomes of the interview.

All data were collected in Danish, and the quotes and extracts presented below have been translated by the lead author of this paper, following the initial analysis.

## 5 FINDINGS

### 5.1 Stage 1

The quantitative analysis of the interviews showed that in all of the interviews, except for one, the interviewees spoke more than the interviewers, illustrating the general asymmetry associated with a good qualitative interview.^21^ In all but three of the eleven joint interviews, the peer interviewers also spoke more than the academic researcher.

A comparison of ARA and API interviews showed that on average, interviewees spoke relatively less in API interviews (65%) compared to ARA interviews (70%), perhaps due to the extra person present in API interviews. However, the API interviews were also slightly longer (on average 16 184 words compared to an average of 13 493 words in ARA interviews) and the average total amount of words spoken by the interviewees in the API interviews was therefore still higher than in the ARA interviews (10 315 and 8234, respectively). The changing dynamics evidenced by the difference in the relative amount of words spoken by each participant in the two types of interviews, thus, has to be considered alongside the total word count, which shows that in general, the interviewees uttered more words in API interviews than in ARA interviews.

The amount of questions and comments posed by the academic researcher and the peer interviewers showed that in the API interviews, the academic researcher had significantly less entries (on average 218 per interview) than the peer interviewers (on average 360 per interview). However, when interviewing alone, the academic researcher had the highest number of entries (on average 418 per interview). An explanation for this is of course that she was the only person interviewing, whereas the API interviews always had two interviewers. The two types of interviews can therefore not be directly compared.

**Figure 1:**
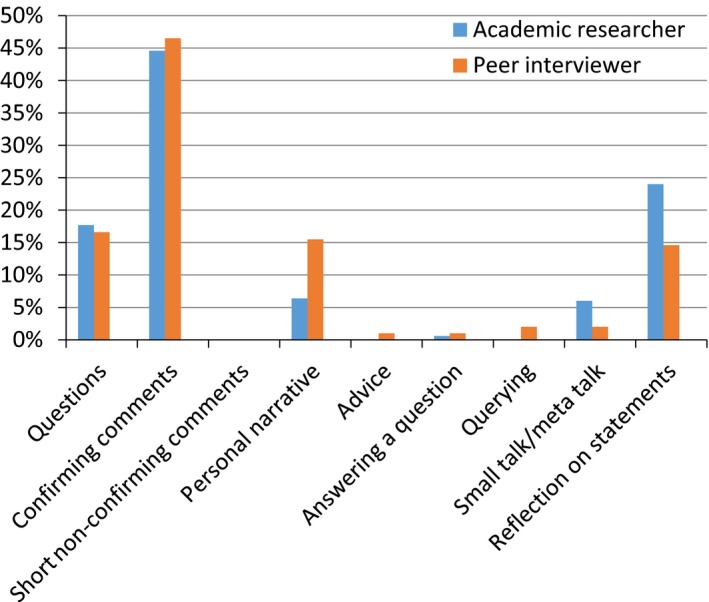
Types of question posed by Academic Researcher and Peer Interviewer (on average in percentages)

The findings from the thematic coding of the entries illustrated some variation in the types of questions or comments posed by the academic researcher and the peer interviewers, respectively (Figure 1). In both types of interviews, “confirming comments” was the type of entry most frequently used by both the academic researcher and the peer interviewers (accounting for 44.6% and 46.5% of all entries). No major differences were found when comparing the use of questions, but there were some differences in the use of personal narratives (with the peer interviewer using this technique more often) and reflection on statements (a technique more frequently used by the academic researcher). A small percentage of the peer interviewer's entries were “advice” and “wondering/querying comments.” These types of entries were not used by the academic researcher, thus showing a slight difference in questioning and interview technique.

**Figure 2:**
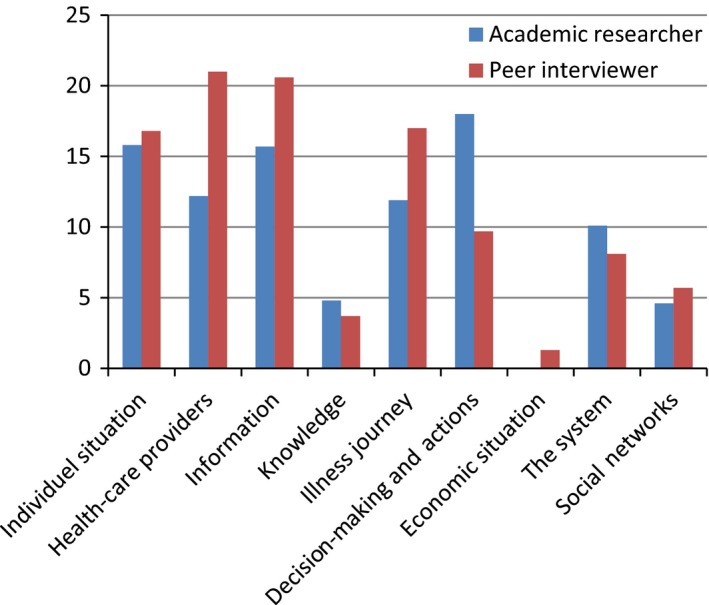
The average percentage of the nine themes in questions asked by the academic researcher and the peer interviewer, respectively

Exercise 3 and 4 divided the questions and the words into nine thematic codes. Following this, the percentage of each code in relation to the total amount of questions/coded text in each interview was calculated and an average of these was found (Figure 2). The thematic coding of the questions showed that even though there were some differences between the academic researcher and the peer interviewers, these were not major. Peer interviewers asked more questions about the individual situation of the patient, their interaction with HCPs, their level of information and their illness journey, whereas the academic researcher asked more questions about decision‐making/actions and slightly more about knowledge and the system.

**Figure 3:**
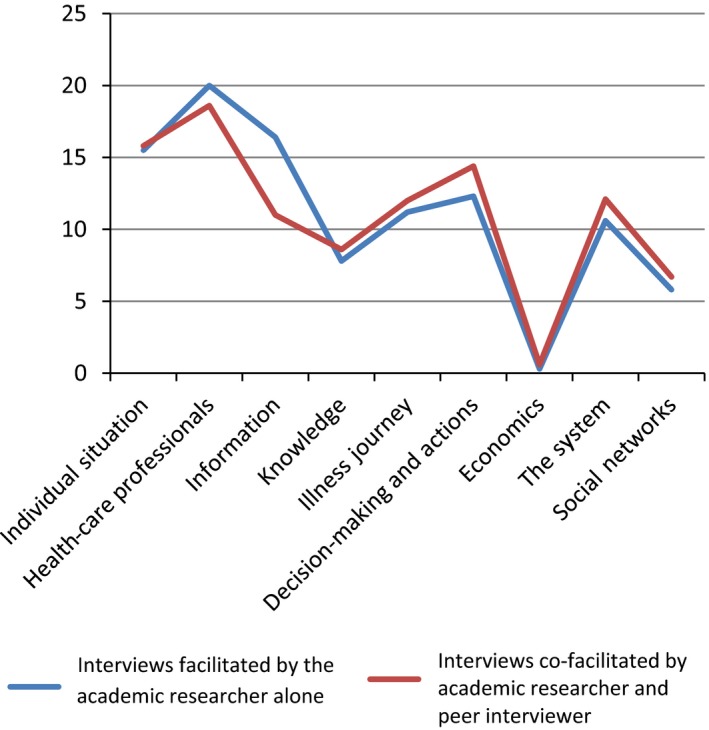
The average amount of words spoken on each of the nine themes in percentages of the total amount of coded words, ARA and API interviews

Along a similar line, the thematic coding of the transcripts as a whole showed no major differences when comparing the two types of interviews. In fact, Figure 3 demonstrated a surprisingly similar pattern, with both types of interviewers focusing mostly on the individual situation of patients, HCPs, information, and decision‐making/actions, less on knowledge, social networks, and not at all on economic situation.

From a purely quantitative perspective, it thus seems that there were no major differences in the amount of questions asked by the academic researcher and peer interviewer and also no significant differences with regard to the relative amount of words used to cover the nine themes when averaging the findings from the two types of interviews.

**Table 1:** The qualitative interviews

Interview number	Interviewee (gender and no.)	Type of interview	Peer interviewer (no. and gender)
1	Female 1	API	1 (Male)
2	Male 1	API	1 (Male)
3	Female 2	ARA	
4	Male 2	API	2 (Female)
5	Female 3	ARA	
6	Male 3	API	1 (Male)
7	Female 4	ARA	
8	Male 4	ARA	
9	Male 4	API	3 (Male)
10	Female 5	API	1 (Male)
11	Female 6	ARA	
12	Female 7	ARA	
13	Female 7	API	4 (Female)
14	Male 5	API	2 (Female)
15	Male 6	API	2 (Female)
16	Female 8	API	4 (Female)
17	Female 9	API	5 (Female)
18	Male 7	ARA	

### 5.2 Stage 2

The qualitative reading of the interviews generally supported the quantitative findings and showed that the peer interviewers tended to insert more information about themselves into the interviews. In many cases, they used their own experiences of illness or of being “in the system” to empathize with the patient or to ask questions, as for example illustrated in the below extracts from interviews:

Peer interviewer: I have also experienced coming into the hospital and being told off for being late. Then I showed the papers and I was, in fact, not late.

Peer interviewer:.. And leading up to this, how was the process? For example with my illness, women can go for up to 6 months with several visits to the doctor without being diagnosed and all that…

Patient: Yes

Peer Interviewer: how did you experience this? Was it quick, or…?

The academic interviewer did not have similar illness experiences to share as part of the interviews or to draw on when phrasing her questions. Instead, she used her experiences from other contexts or her knowledge of what other people had said to create a sense of commonness or understanding:

“I think this is my interview no. 11 [and] this is one of the dilemmas I have encountered. It is really complex”

Both types of interviews generally had a good flow and the interviewed patients responded well to the questions asked. The process of trying to identify any potential difference between the two sets of interviews and assess whether the presence of peer interviewers and the different levels of personal narrative included in the interviews had any effect on the interviewee and the research outcome was difficult. Even though the research design was constructed, so that the two types of interviews could be compared, the unique, socially constructed, and context‐dependent characteristic of qualitative interviews^22^ made it very difficult to evaluate whether any particularly informative interviews were due to the interviewers, the interviewee, or the dynamics between them. However, what became increasingly clear through the reading of the transcripts was that not only were there some differences between the academic researcher and the peer interviewers with regard to the extent of information they inserted into the interview, the peer interviewers also varied significantly amongst themselves. This illustrates the importance of considering the variety of peer interviewers and the potentially different impact they may have on research process and outcome.

### 5.3 Stage 3

The analysis of the notes from the phone conversations with the interviewees showed that they were generally very positive about having a peer interviewer present. When asked what their initial reaction had been, several of them mentioned that they had thought it was a good idea to have someone in the interview with similar personal experiences and that they had expected it would bring a “patient‐perspective” or “user‐perspective” to the interview.

“She (the peer interviewer) can provide some prompts – input to open up your memories”

“The academic researcher ‐ she was more professional… the other one, it was based on her own experiences”

One participant mentioned that she had been concerned about how the previous experiences of the peer interviewer might impact on the interview:

“When someone has been struck by cancer, when you talk about it, they hear their own situation, and not the situation of the person in front of them. It takes a long time to understand, that I don't feel like that”

However, when reflecting on the interview itself, she added that her concerns had not materialized, as the peer interviewer had shown good listening skills and focused on the experiences of the interviewee rather than her own.

While the interviewees thus generally had felt that it had been a good experience to have a peer interviewer present, they varied in their opinion as to whether it had made a difference to the outcome of the interview. A common theme in the interviewees' narratives was that they had often told their story many times, or that they were generally very open about their experiences, and therefore not in need of any particular kind of interviewer to facilitate conversation. However, one male interviewee mentioned that it was very positive to have a peer interviewer present, because “he [the peer interviewer] knew what sort of questions to ask and because it made you think in new directions.” In addition, he mentioned gender as a contributing factor to the (female) academic researcher being a “bit out” during the interview. However, another male interviewee did not seem to consider gender as a major issue. He mentioned that the peer interviewer had thought of new questions and that her knowledge in the area had made her a “good partner” in the interview. Gender might therefore be more important in some situations than others.

Finally, two interviewees mentioned that the peer interviewer who had conducted their interview had had a health professional background and that this had been a benefit for the interview and the interviewee him/herself, as the peer interviewer had provided new knowledge and ideas, and thus made the interview a learning process, also for the interviewee. As this illustrates, peer researchers may draw on other experiences besides their personal ones and this may bring up some dilemmas with regard to managing the boundaries between being a researcher, a peer and a professional.

## 6 DISCUSSION

In our attempt to evaluate whether any differences could be identified in the *proces*s of carrying out the interviews (research question 1) and in the actual *outcomes* (research question 2) of the Empowerment study, we adopted a multifaceted approach, including both quantitative and qualitative analysis.

Some quantitative differences were identified in the way the academic researcher and the peer interviewers asked questions and the comments they provided. These differences were supported by the qualitative reading of the interview transcripts which showed that peer interviewers generally inserted more information about themselves into the interviews than the academic researcher, but that the different types of peer interviewers also varied widely in their approach. The academic researcher did not have any personal experiences of illness to bring to the interview, but drew on other experiences to develop a sense of “commonness.” Without negating the power of shared illness experiences, these findings illustrate the importance of considering similarities and differences at a range of levels^23^ and open up for a broader discussion of insider/outsider perspectives in peer interviews. As noted by Thomson et al^13^ and Greene et al,^24^ it is often highly complex to determine what constitutes a “peer,” as peer interviewers may have certain characteristics in common with their interviewees, but may also differ significantly in others. In assessing the impact of peer interviewers on the process of the interview, it is therefore important to critically consider other characteristics, besides the one(s) the peer interviewers have in common with their interviewees. For example, as previously noted, shared gender was mentioned by one of the interviewees in this study as having contributed positively to the interview process and in other studies other characteristics may have a similar effect.

Considering the insider perspective also brings up some important ethical dilemmas with regard to shared emotions and the potential impact on the peer interview process. One of interviewees expressed feelings of compassion for the peer interviewer due to his advanced stage of illness and due to his own memories of being afraid of the same situation. While he did not believe that this had affected the interview itself, he did mention that it had affected him emotionally. As this shows, the practice of involving peer interviewers poses some important questions about the emotional impact on already potentially vulnerable interviewees or their interviewers.^12^ While the interviewees in the Empowerment study generally focused on the positive impact of shared experiences, it is thus also important to consider the risk of any potential distress these may bring.

Summarizing the findings in relation to questions one, it can thus be argued that the personal narratives used by peer interviewers in the Empowerment study and the experiences they shared with the research participants seemed to have some impact on the process of the interviews and the way the participants experienced it.

Our second question asked whether any impact could be discerned on the outcome of the interviews. While the word counts performed as part of the first stage of analysis showed that the API interviews were generally longer and may have collected more data in total, the topics covered by the questions followed a relatively similar pattern. The coding of the interview content furthermore showed that the percentages of words used to discuss the nine thematic codes were surprisingly similar, leading to the conclusion that in the Empowerment study, the academic researcher and the peer interviewers were relatively well‐coordinated and obtained similar outcomes.

However, the averages used to get a relative idea about the amount of words spent to discuss each code may have covered up potentially large differences between interviews. In addition, the nine thematic codes used for the analysis were very broad, and did not allow us to assess whether for example more intimate or personal aspects of the themes were discussed when a peer interviewer was present.

Acknowledging these concerns, we tried to explore some of the individual differences in the qualitative reading of the transcripts. This was, however, much more difficult than first anticipated, mostly because qualitative interviews are essentially unique and therefore very difficult to compare. Any differences between them had to be considered in light of a number of variables, including not only whether or not there was a peer interviewer present at the interview, but also considering gender and illness similarity, the experiences of the interviewees in the health system, and their particular personality and narrative style.

The phone conversations with interviewees were also not able to bring any firm conclusions about the impact of peer interviewers on the outcome of the interviews. In general, interviewees had been happy about the process of having peer interviewers in the interviews, but none of them could say whether the outcome would have been different had they not been present. One of the participants did mention that the peer interviewer had been able to bring knowledge to the interview, making it a learning process for both interviewer and interviewee. While this comment suggests a certain positive impact on the research outcomes, it also poses a number of questions with regard to the accuracy of the knowledge passed on, and the distinction between professional and personal research conduct. As discussed by Dickson‐Swift et al,^18^ blurred boundaries between professional and personal is an issue often encountered in qualitative health research, particularly on sensitive subjects. These boundaries may be further challenged in peer research, where interviewers have been recruited specifically because of their personal experiences, and may therefore identify strongly with their research participants.^24^ However, even though the peer interviewers in the Empowerment study did use their personal experiences as the basis for the interviews, the qualitative reading of the interview transcripts did not point towards any excessive guiding of the interviewees towards particular topics or perspectives.

## 7 CONCLUSION AND PERSPECTIVES

This article has discussed and explored the impact of peer interviewers in the Empowerment study, using a three‐staged data analysis process. This process showed that peer interviewers generally tended to insert more information about themselves into the interviews, but also varied significantly in their approaches. Any firm conclusions on whether this had an impact on the outcomes of the interviews were not possible to make.

The study found that interviewees were generally content with the process of having a peer interviewer present, and some had felt that being interviewed by someone with similar experiences had been very useful. However, their comments also highlighted some potential issues when having peer interviewers, particularly the emotional impact, and the sometimes blurred boundaries between the personal and the professional. In addition, the responses highlighted that what makes someone a peer can be different in different situations, and that peer interviewers may also draw on other experiences than the ones they have in common with their interviewees.

Based on the analysis presented in this paper, we find that there are good arguments for using peer interviewers in qualitative health research, although the extent of the benefits may depend on the topic of the research, its participants and its particular context. In any study, it is furthermore important to consider potential benefits alongside relevant ethical considerations, available resources for support of both peer interviewers and interviewees, and the need for training, not only in interview techniques, but also in reflexivity and professional/personal boundary work.

## CONFLICT OF INTEREST

The authors declare no conflict of interests.
